# Facial cutaneous Rosai-Dorfman disease: a case report

**DOI:** 10.1186/s13256-024-04410-9

**Published:** 2024-03-27

**Authors:** Tanapong Wongrat, Siripan Sangmala

**Affiliations:** 1 Division of Dermatology, Department of Internal Medicine, Faculty of Medicine, Prince of Songkla University, Songkla, 90110 Thailand; 2 Department of Internal Medicine, School of Medicine, Mae Fah Luang University, 365 Tambon Nang Lae, Amphoe Mueang Chiang Rai, Chang Wat Chiang Rai, Chiangrai, 57100 Thailand

**Keywords:** Cutaneous Rosai-Dorfman, Skin, Hematology, Histiocytosis, Case report

## Abstract

**Background:**

Rosai-Dorfman disease (RDD) is a form of non-Langerhans cell histiocytosis in which the activated histiocytes of the lymph nodes and other organs begin to accumulate following excessive production. Bilateral, massive, and painless lymphadenopathy are classic presentations. Systemic RDD is already known to be a rare condition, but isolated cutaneous RDD is extremely rare. We presented a rare and unusual presentations of a disease.

**Case presentation:**

A 35-year-old Thai female with a 6-month history of a small acne-like lesion that rapidly progressed to 5 cm tumor-like lesions on the face within 3 months. Tissue histology showed a dense dermal infiltration of histiocytes with emperipolesis phenomenon. Immunohistochemistry was positive for S100 protein and CD68 and negative for CD1a. Oral prednisolone (50 mg/day) was initiated with a favorable outcome at the one-month follow-up. However, prednisolone yielded a partial response at 2-month follow-up, leading to application of another modality.

**Conclusion:**

Although cutaneous Rosai-Dorfman disease is considered benign and well medical responded disease, patients with atypical presentation and rapid growing lesion may necessitate aggressive multimodal treatment.

## Background

Rosai-Dorfman disease (RDD) is a form of sinus histiocytosis characterized by massive lymphadenopathy. It occurs when non-Langerhans sinus histiocytes are produced to excess and begin to accumulate in the lymph nodes and other organs. It is a rare and often unrecognized disease, with patients commonly presenting with bilateral, massive, and painless lymphadenopathy with which extranodal forms were reported in 43% of cases [1]. The most common extranodal site is the skin [2]. Although 10% of cases were reported to have skin involvement, only 3% had the pure skin manifestation called cutaneous Rosai-Dorfman disease (CRDD). The face was the most common site of cutaneous involvement, with a combination of varying skin morphologies and discoloration [3]. The overall prognosis of the disease is good with an indolent clinical course, and the availability of multiple treatment modalities [1]. We reported a rare case of facial CRDD with an unusual manifestation.

## Case report

A 35-year-old Thai female presented with a 6-month history of an asymptomatic single, small acne-like lesion on her left cheek (a 1.5 cm red-yellow nodule). She had previously seen a general practitioner and was transferred to the nearby dentist to check her dental condition that might have caused the lesion. Despite taking off her dental caries, the facial lesion rapidly grew in size with a few new small erythematous papules occurring. She reported no fever, no poor appetite, no night sweats, nor weight loss. She had no underlying disease and denied taking herbs, supplements, or other oral medication. None of her family members reported the same clinical entity.

**Fig. 1:**
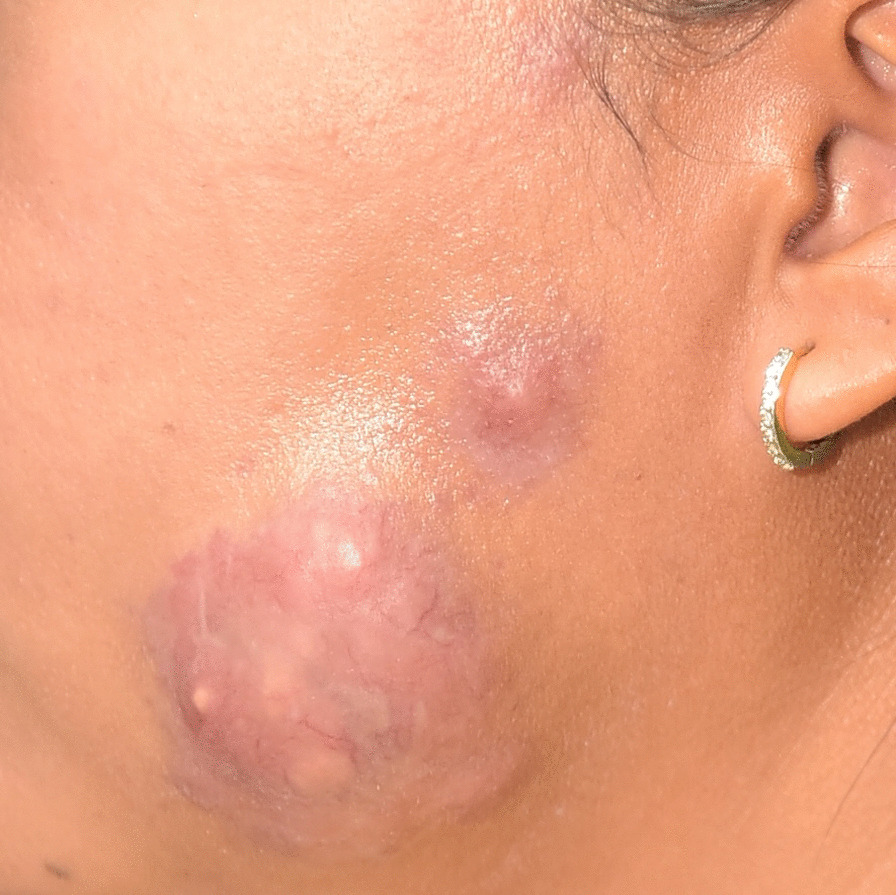
A reddish-yellow tumor-like lesion, measuring 5 cm in diameter with a single small nodular lesion on the left face

**Fig. 2:**
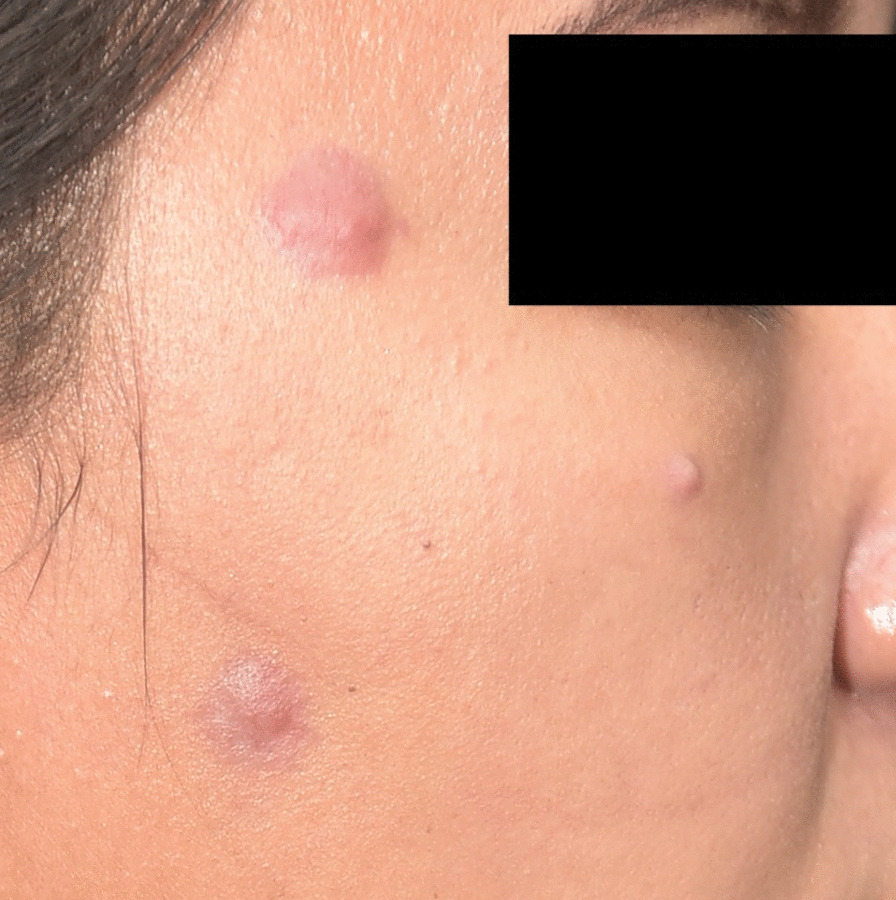
Few 1–1.5 cm erythematous papules on the right face

The examination showed a reddish-yellow tumor-like lesion on her left cheek, measuring 5 cm in diameter, (Fig. 1) with a few 1–1.5 cm erythematous papules scattered on the face (Fig. 2). The rest of the general physical examinations were normal, without lymph node enlargement or hepatosplenomegaly.

**Fig. 3:**
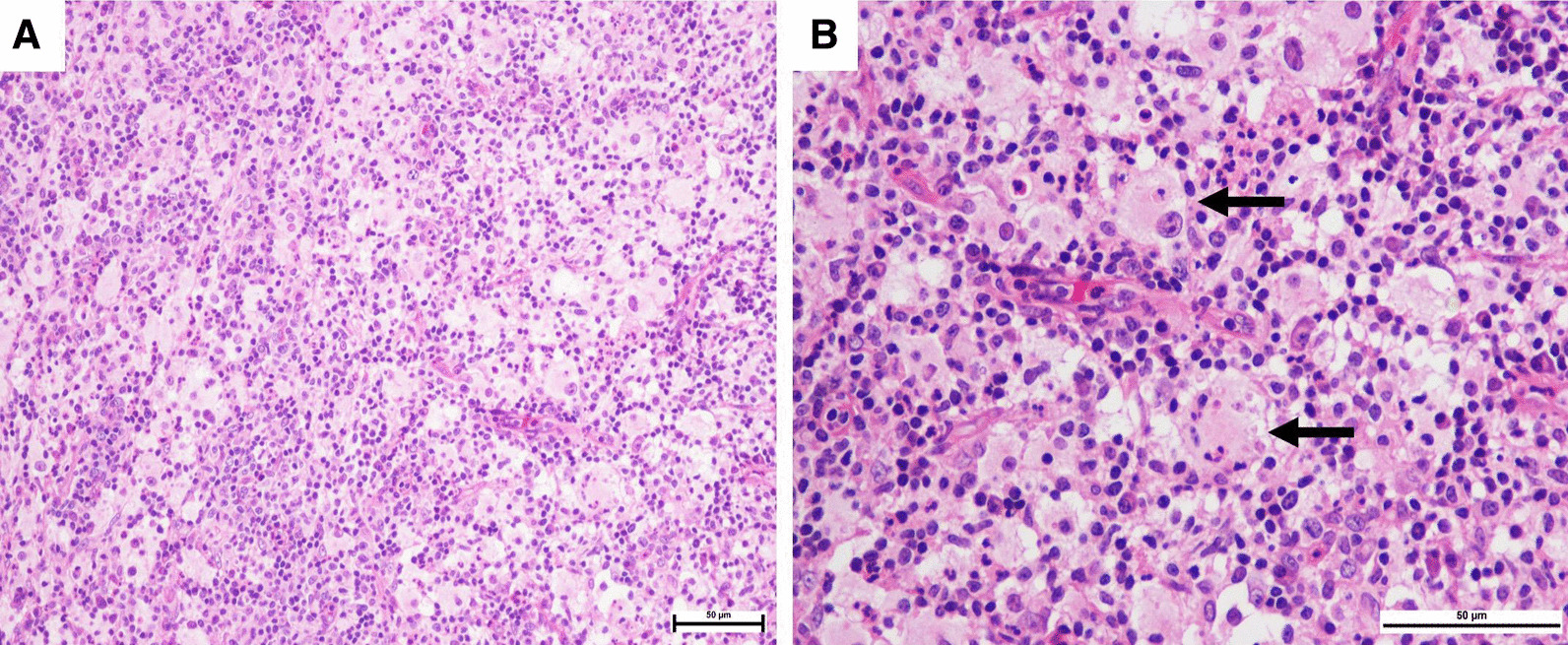
**A** Histiocytes with prominent round nuclei and abundant pale cytoplasm in combination with some small lymphocytes, plasma cells, and scattered neutrophils; **B** Engulfment of lymphocytes into cytoplasm or emperipolesis (black arrows). (Hematoxylin and eosin, original magnification × 200 (A); × 400 (B))

**Fig. 4:**
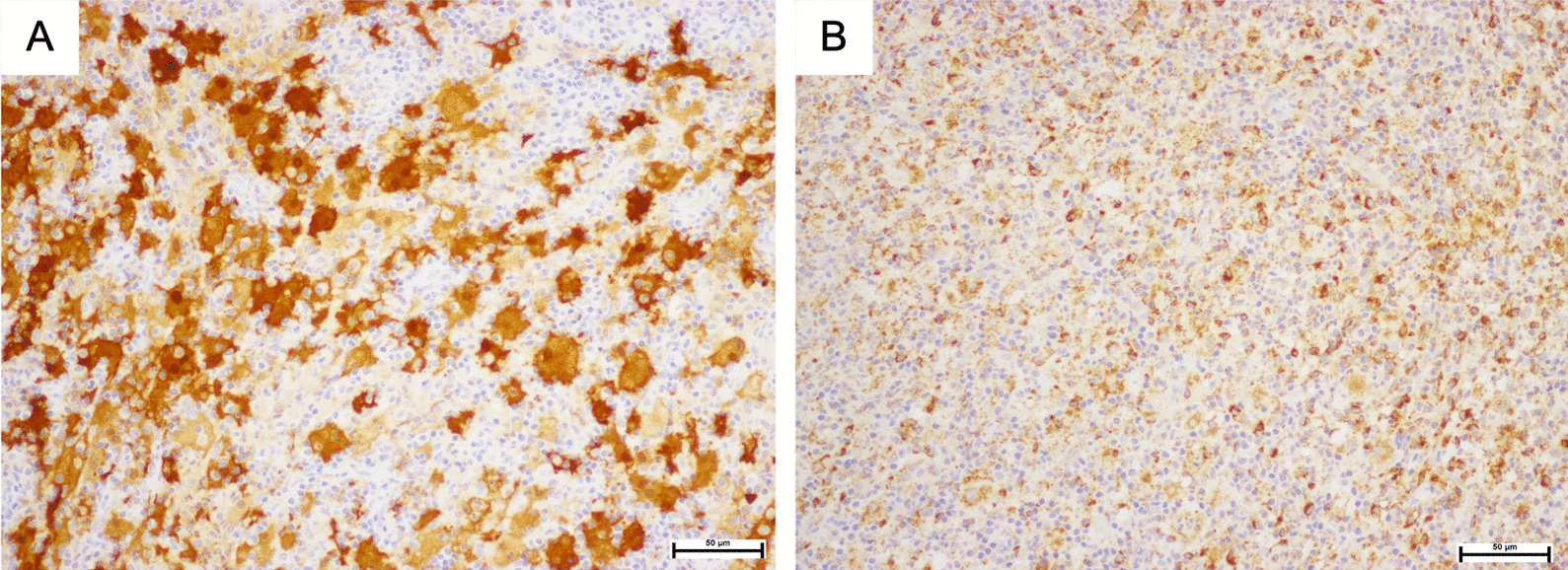
**A** Histiocytes were positive for S-100; **B** Histiocytes were positive for CD68 + (SP staining, original magnification ×200 (**A**); CD68, original magnification ×200 (**B**))

**Fig. 5:**
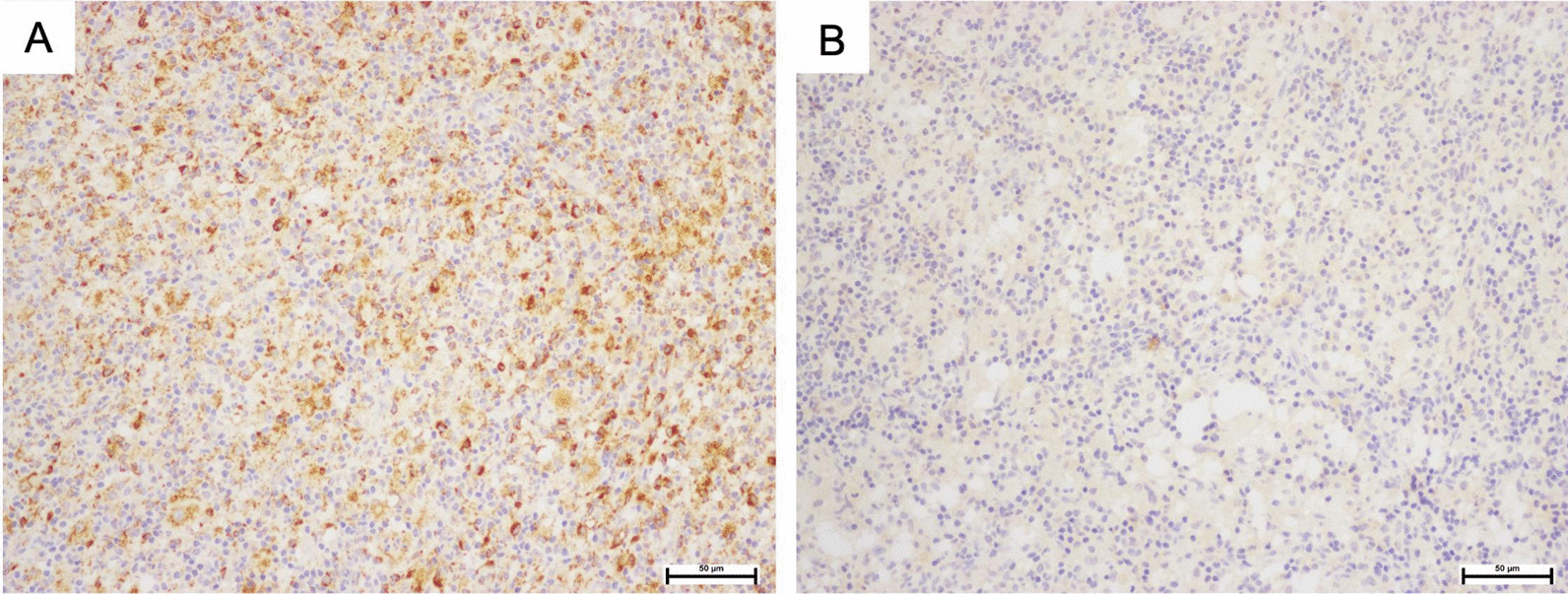
**A** Nuclei of histiocytes were positive for cyclinD1; **B** Negative CD1a in histiocytes (cyclinD1, original magnification ×200 (**A**); CD1a, original magnification ×200 (**B**))

Tissue histology was obtained by a punch biopsy and revealed a dense dermal infiltration of mixed inflammatory cells, prominently of histiocytes with large vesicular nuclei and pale voluminous cytoplasm, admixed with some small lymphocytes, plasma cells, and scattered neutrophil without eosinophil (Fig. 3A). Engulfment of intact lymphocytes, or the emperipolesis phenomenon, was observed (Fig. 3B). Immunohistochemistry showed positive for S100 protein, CD68, and cyclinD1 + indicated histiocytes, and negative for CD1a indicated non-Langerhans cells (Figs. 4A, B, 5A, B). Computed tomography scans of the brain, neck, chest, and whole abdomen revealed a non-significant 0.8 cm of the cervical lymph node. The bone marrow study was negative for malignancy. Basic laboratory tests were also normal. However, molecular genetic testing was limited in our hospital.

The patient was treated with oral prednisolone (50 mg/day). At the one-month follow-up, the lesion had decreased in size giving a favorable outcome. However, prednisolone resulted in a partial response at the two-month follow-up, so methotrexate 25 mg/wk was added. As of now she is undergoing treatment, including a taper dose of steroids (15 mg/day) and methotrexate 25 mg/wk, with regular and monthly follow-ups.

## Discussion and conclusion

RDD can be categorized under histiocytes and neoplasms of the macrophage-dendritic cell lineage; it is an idiopathic, benign non-Langerhans histiocytosis. It can be found in two different forms. The first is classical RDD, which usually involves the lymph nodes and very occasionally the extranodal organs, exhibiting systemic symptoms. The second type is solely CRRD [4]. The two forms differ in their clinical presentation and diagnosis. The prevalence of classical RDD is very low, at around 1 in 200,000. Annually, just 100 new cases are found. Skin lesions are present in around 10% of classical cases, but only 3% of cases are classified as purely CRDD. However, in the recent study, the prevalence of isolated CRDD reached as high as 18% [2]. Classical RDD is often reported in children and young adults (mean age 20.6 years), predominantly in males and those of African ethnicity, while CRDD commonly affects adults (mean age 43.7 years) and Asian females [5]. Our patient was a 35-year-old Thai woman who was therefore compatible with the CRDD epidemiological pattern.

It is common for classical RDD patients to exhibit the symptoms of bilateral, massive, and painless lymphadenopathy, sometimes combined with extranodal involvement. CRRD may exhibit in isolation or in combination with both nodal and other extranodal involvements. Frequently observed symptoms on the skin include non-pruritic, dark red or purple papulonodular lesions or plaques which are usually painless and grow slowly. These are found most commonly on the face and trunk, and sometimes on the extremities [6]. The work of Kong et al. examined 25 CRDD patients from China who presented with 39 lesions, categorizing the skin morphologies into three principal forms: papulonodular (79.5%), indurate plaque (12.8%), and tumors (7.7%) [7]. Accordingly, the condition of our patient is very rare, and for isolated CRDD, the progression observed is not typical, as the reddish-yellow tumor-like lesion is growing very quickly.

The pathogenesis of CRDD is not well established; it may be associated with viral infection such as the human herpes virus, Epstein-Barr virus, cytomegalovirus, or HIV [8]. Moreover, recent studies demonstrated that mutations of NRAS may be related to CRDD [9]. Our patients reported no history of previous viral infection, however genetic testing is limited in our hospital.

From a histopathological point of view, classical RDD and purely CRDD share similar findings. Histiocytes with ample pale or “watery-clear” cytoplasm with a hypochromic nucleus and distant round nucleolus. The emperipolesis phenomenon, defined as an engulfment of intact leukocytes and inflammatory cells by the histiocyte cytoplasm, is a helpful presentation, but is not required for diagnosis. In addition, a heterogenous background of lymphocytes, plasma cells, and neutrophils without eosinophils are commonly observed. Immunohistochemistry is typically positive for S100, CD68, CD163, and CD14, while negative for CD1a and CD207 [10]. Some studies reported differences of histology in RDD and CRDD, in that CRDD has more fibrosis and sclerosis, and less histiocytes and emperipolesis. These features are helpful in ruling out other differential diagnoses. For examples, Langerhans cell histiocytosis is positive for CD1a and CD207, and commonly has eosinophilic accumulation. In xathogranuloma, non-Langerhans histiocytosis, the absence of emperipolesis phenomenon is a helpful manifestation [11].

RDD is a benign disease with spontaneous remission and responds well to medication in most cases. CRDD can be treated via various approaches. The American Society of Hematology in 2008 sought to establish a common standard governing both the diagnosis and subsequent management of Rosai-Dorfman cases. However, there is no uniform approach for treatment, so the decision is based on the individual clinical circumstances. Around 20–50% of patients with nodal/cutaneous RDD can experience spontaneous remission, thus observation is appropriate for asymptomatic or uncomplicated cases. Surgical resection is a curative treatment for small unifocal lesions. A systemic or local form of corticosteroid is commonly prescribed as a first line medication but results in variable outcomes. Other modalities include sirolimus, radiotherapy, chemotherapy (methotrexate, 6-mercaptopurine) and immunotherapy (thalidomide, rituximab), while targeted therapies (imatinib) are suitable for more severe, refractory cases or life-threatening conditions [1]. For our case, we planned to initiate with oral prednisolone 1 mg/kg/day with a combination of intralesional corticosteroid in the case of an unfavorable outcome from prednisolone, and finally, a surgical approach for curative treatment when the lesion is small and appropriate for resection. Our patients had satisfactory outcomes at the one-month follow-up after starting prednisolone; however, the lesions were not improved at the two-month follow-up, thus low dose methotrexate (25 mg/week) was added to the protocol. Now she was on treatments with regular monthly follow-ups.

In conclusion, we presented a case with a rapidly growing large tumor type of purely CRDD on the face, which is an unusual manifestation, and the lesion progression made diagnosis difficult. However, histopathological findings and immunohistochemistry staining were compatible with CRDD, and were essential to diagnose CRDD and distinguish the condition from other diseases. Despite CRDD being a benign and self-limited disease, patients with atypical presentation and rapid progression may need aggressive, and multimodal treatment.

## Data Availability

All information is available in the clinical records archive of the Songklanagarind Hospital, Prince of Songkhla university, Songkhla, Thailand.

## Abbreviations

RRD: Rosai-Dorfman disease
CRRD: Cutaneous Rosai-Dorfman disease
HIV: Human immunodeficiency virus
